# Inequality in dental flossing behavior among Korean adults based on household income levels

**DOI:** 10.4178/epih.e2024052

**Published:** 2024-05-24

**Authors:** Zi-Lan Wang, Eun-Jae Choi, Seung-Hee Ryu, Seon-Jip Kim, Hyun-Jae Cho

**Affiliations:** 1 Department of Preventive Dentistry & Public Oral Health, and Dental Research Institute, Seoul National University School of Dentistry, Seoul, Korea; 2 Dazhou Vocational and Technical College, Dazhou, China

**Keywords:** Dental devices, Income, Oral health, Socioeconomic disparities in health

## Abstract

**OBJECTIVES:**

The aim of this study was to estimate the association between household income and dental flossing.

**METHODS:**

This cross-sectional study investigated the impact of household income on flossing among 9,391 adults aged 30+ with ≥20 natural teeth, utilizing data from the seventh Korea National Health and Nutrition Examination Survey (2016-2018). Outcome measures included flossing (yes/no), with income categorized into 4 levels: lowest, medium to low, medium to high, and highest. Logistic regression, adjusted for age, gender, brushing frequency, recent dental exams, periodontitis, smoking, and alcohol use, was employed to evaluate the influence of socioeconomic status on oral hygiene practices.

**RESULTS:**

In the highest income group, flossing was 62.6% more prevalent than in the lowest income group (adjusted odds ratio [aOR], 1.63; 95% CI, 1.27 to 2.08). The strongest association between income levels and flossing was observed in individuals aged ≥70 years (aOR, 3.64; 95% CI, 1.86 to 7.11), with a decreasing strength of association in the 60s (aOR, 1.72; 95% CI, 1.05 to 2.84) and 50s age groups (aOR, 1.69; 95% CI, 1.07 to 2.68). Higher-income women demonstrated a higher frequency of flossing than their lower-income counterparts (aOR, 1.67; 95% CI, 1.24 to 2.23). Higher-income individuals without periodontitis were more likely to floss (aOR, 1.64; 95% CI, 1.23 to 2.18), and among those with periodontitis, flossing was significantly associated only with the highest income category (aOR, 1.64; 95% CI, 1.10 to 2.44).

**CONCLUSIONS:**

The findings of this study indicate a significant correlation between higher household income levels and an increased prevalence of flossing.

## GRAPHICAL ABSTRACT

[Fig f3-epih-46-e2024052]

## Key Message

This study highlights that the use of dental floss is significantly influenced not only by personal health behavior habits but also by socioeconomic status. Individuals in higher income classes tend to use dental floss more frequently, which is associated with higher levels of education, health literacy, and economic resources. Public health planning should take economic factors into account and focus on improving dental hygiene education and access to dental care products.

## INTRODUCTION

Dental plaque is a sticky biofilm that forms on teeth and is a primary cause of major dental diseases, such as dental caries and periodontal diseases [1,2]. The primary method for controlling the accumulation of supragingival plaque is the mechanical removal of dental plaque [3]. The most common technique for mechanical dental plaque control is toothbrushing [4]. Although toothbrushing is fundamental to oral hygiene, its effectiveness is limited by the user’s technique and the toothbrush’s ability to access all tooth surfaces. Specifically, toothbrushes are less effective at removing plaque from interproximal surfaces [5]. In addition to toothbrushing, dental floss, when used as a supplementary aid, can effectively target the difficult-to-reach areas between teeth and help reduce gingival inflammation [6]. Gingivitis and periodontal disease are the second most commonly reported outpatient diseases in Korea, leading to a significant burden of periodontal disease for many people. This burden results in the loss of social resources and requires substantial financial resources [7,8]. The use of oral care products is a behavior that can prevent gingivitis and periodontal disease, potentially reducing oral health inequalities that arise from the treatment of oral diseases due to varying income levels [9]. The American Dental Association reports that using dental floss may remove up to 80% of dental plaque and recommends its use at least once daily [10]. However, various factors contribute to the persistently low rates of dental floss use [11,12]. Notably, findings from a cross-sectional study spanning 12 years indicate that more than 80% of Korean adults over the age of 30 do not engage in flossing [13].

Socioeconomic disparities in oral health are a major public health concern worldwide [14]. The relationship between individual or household income and oral health status has garnered considerable attention. However, the relationship between income level and oral health behaviors, particularly the use of dental floss, has not been extensively explored. Research conducted in the United States has revealed substantial socioeconomic disparities across most oral health-related behaviors [15]. This correlation highlights the influence of economic status on oral health behaviors and practices. It can be inferred that individuals with greater financial resources not only have better access to dental care services but may also possess a higher awareness and capability to maintain oral health behaviors, such as using dental floss.

A previous cohort study conducted in Iran reported socioeconomic inequalities in flossing [16]. However, that study focused solely on socioeconomic status and education levels, neglecting potential confounding factors such as the frequency of daily toothbrushing, the conduct of oral examinations, periodontitis, smoking, or alcohol consumption. By taking these factors into account, we can more accurately determine whether a true association exists between flossing and economic status.

Based on current knowledge, the issue of socioeconomic inequalities in dental flossing among Korean adults warrants further investigation. We hypothesized that flossing is more prevalent in households with higher income levels, reflecting socioeconomic disparities in oral health behaviors. Therefore, the objectives of this study were to assess the impact of household income on the rate of dental flossing in the Korean adult population aged over 30 with 20 or more natural teeth, using data from the seventh Korean National Health and Nutrition Examination Survey (KNHANES VII).

## MATERIALS AND METHODS

### Data and study population

**Figure 1.**
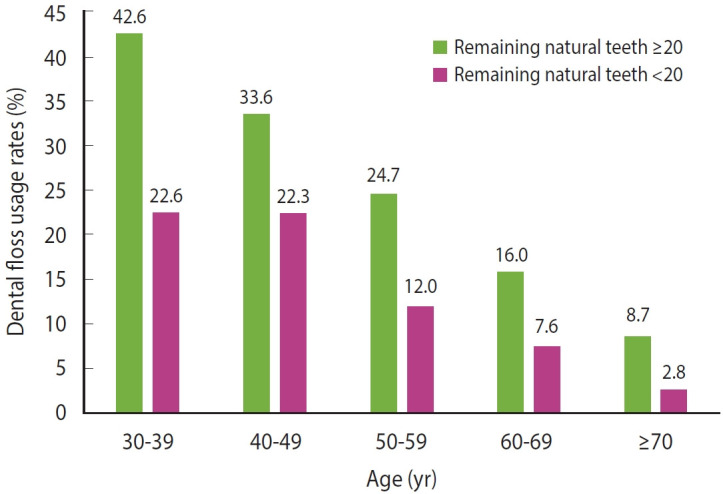
Age group variation in dental floss usage according to number of natural teeth.

This study analyzed data from the KNHANES VII. The KNHANES is a cross-sectional, nationally representative survey designed to assess the health and nutritional status of the Korean population [17]. The sampling framework employed a multistage clustered probability design, selecting participants from 576 districts across Korea to ensure comprehensive representation of the population [18]. Only individuals with 20 or more teeth were included in this study, as the number of teeth affects their spacing and arrangement, which in turn influences flossing. For individuals with 20 or more teeth in the oral cavity, dental flossing may significantly prevent periodontitis [19]. In this study, the rate of flossing was higher among participants with ≥ 20 remaining teeth than in those with < 20 remaining teeth (Figure 1). The study initially included 9,459 individuals, aged 30 years and over and with 20 or more natural teeth. In studies utilizing data from the KNHANES that focus on income-related aspects, research participants are frequently selected from the age group of 30 years and above [13,20]. This selection criterion is based on the understanding that individuals below this age threshold may not yet have established their own income sources and typically exhibit better oral health outcomes. Thus, the data are typically restricted to this older age group, enabling more dependable inferences about the relationship between income status and oral health. Nevertheless, due to incomplete or missing data concerning flossing, 78 participants were excluded from the analysis. As a result, the final cohort for the study was composed of 9,381 participants.

#### Outcome variable

Flossing was the primary outcome measured in this study. Dental flossing habits were assessed using self-reported data obtained from the survey participants.

#### Explanatory variable

Household income was the explanatory variable. We utilized the monthly average equivalized household income quartile for each year as the classification criterion due to differences in income across the survey years [21]. The categories were defined as lowest, medium-low, medium-high, and highest.

#### Covariates

The covariates in the analysis included socio-demographic factors, personal health practices, and medical status. The socio-demographic factors analyzed included age (continuous, years) and gender (man or woman). Personal health practices were assessed based on the frequency of daily toothbrushing (categorized as ≤ 1 or ≥ 2 times/day) and whether oral examinations had been conducted in the past year (categorized as yes or no). Medical status variables included the presence of periodontitis (categorized as yes or no), smoking status (categorized as current, past, or never), and alcohol consumption (yes or no).

### Statistical analysis

Age was treated as a continuous variable, while other variables were analyzed as categorical variables. Descriptive statistics are presented as weighted percentages (%) and 95% confidence intervals (CIs), derived from complex sampling methods. A chi-square analysis was conducted to examine variations in dental flossing habits across different ages and household income groups. To investigate the effect of household income on dental flossing, logistic regression within a complex sample design was employed. We analyzed several models, each adjusting for different covariates (model 1: age and gender; model 2: oral examinations in the past year, daily toothbrushing, and periodontitis; model 3: smoking and alcohol consumption). These adjustments aimed to mitigate potential confounding effects on the relationship between household income and flossing frequency. Furthermore, we stratified the data by age group, gender, and periodontal health status and applied a complex-sample logistic regression model to evaluate the relationship between household income and the rate of flossing. The threshold for statistical significance was set at p<0.05. All statistical analyses were conducted using SPSS version 26 (IBM Corp., Armonk, NY, USA).

### Ethics statement

The study protocol, including the use of human participants and ethical considerations, was reviewed and approved by the Research Ethics Committee of the Korean Disease Control and Prevention Agency (KDCA). Approval for this study was granted under number ‘2018-01-03-P-A’. All respondents provided written informed consent.

## RESULTS

The study included 9,391 adults aged 30 and older, comprising 3,983 men and 5,398 women, as per the unweighted participant count. Table 1 offers a comprehensive description of the study population, detailing demographics and various confounding variables, and categorizes the data based on flossing. The analysis revealed a clear gradient in flossing behavior associated with household income among individuals aged 30 and above who had more than 20 natural teeth. Those in the highest income bracket showed a flossing rate of 10.6% (95% CI, 9.6 to 11.6), which was significantly higher than the 2.0% rate (95% CI, 1.6 to 2.3) recorded in the lowest income bracket. Flossing rates in intermediate income levels increased progressively; the medium-low and medium-high income groups reported flossing rates of 5.9% (95% CI, 5.3 to 6.5) and 9.1% (95% CI, 8.4 to 9.9), respectively.

Table 2 presents the multivariate association between household income levels and dental floss use rates, providing odds ratios with 95% CIs across 3 models. All results were significant, with a p-value of less than 0.05. In the simplest model (model 1), the highest income level was associated with nearly double the odds of using dental floss compared to the lowest income level, with an adjusted odds ratio (aOR) of 1.92 (95% CI, 1.51 to 2.44). Models 2 and 3 included additional variables and controlled for potential confounding factors. Despite these adjustments, the trends remained consistent. The highest income group’s aOR decreased slightly to 1.65 (95% CI, 1.30 to 2.11) in model 2 and to 1.63 (95% CI, 1.27 to 2.08) in model 3. Nevertheless, a significantly higher likelihood of using dental floss among the higher income groups persisted. The medium-high and medium-low income groups also demonstrated a statistically significant association with flossing in both adjusted models.

Based on the significant aORs shown in Table 3, this study identified compelling associations between household income levels and flossing across various demographic categories and periodontal statuses. Notably, within the 50-59-year age group, individuals in the highest income bracket were 69% more likely to use dental floss (aOR, 1.69) than those in the lowest income bracket. This association was even more pronounced among older adults (≥ 70 years), where individuals in the medium-high and highest income brackets showed nearly triple and double the likelihood of using dental floss, respectively (aORs up to 3.64).

Significant gender differences were also observed, with women demonstrating a stronger and more consistent association between higher incomes and flossing across the medium-high and highest income brackets (aORs, 1.64 to 1.67). Thus, women with higher incomes were more likely to use dental floss than those with lower incomes.

Regarding periodontal health status, individuals without periodontitis and with higher incomes were more likely to use dental floss, as indicated by significant aORs of 1.41, 1.66, and 1.64 across different income levels. Conversely, among individuals with periodontitis, a significant association was only found at the highest income level, with an aOR of 1.64, corresponding to a 63.7% higher likelihood of flossing.

## DISCUSSION

Our study indicates that the use of dental floss is not solely a matter of personal hygiene habits but is significantly influenced by socioeconomic status, with individuals in higher income brackets more likely to use dental floss. This disparity underscores the importance of public health initiatives aimed at improving oral health literacy and making oral care products more accessible across various income levels. By expanding access to and education about oral health care, especially among lower-income groups, we can help reduce the observed inequalities in flossing and, consequently, in overall oral health outcomes. Reflecting on the broader implications of our findings, it is clear that addressing these disparities requires a comprehensive approach, as supported by our study and corroborated by existing research. Our results align with previous studies [16] that have shown that individuals with higher incomes are more likely to use dental floss than those with lower incomes, a pattern that persists even after adjusting for potential confounding factors. To date, this study is among the few that have assessed the relationship between household income level and flossing in the Korean adult population. Evidence from multiple sources indicates that the distribution of dental care utilization varies across countries and socioeconomic groups [22-24]. Research findings related to income inequality and oral health have consistently demonstrated a significant disparity in flossing [25,26]. Based on our findings and the literature, we have identified 4 potential reasons why household income levels might influence flossing.

The first factor to consider is the direct cost of purchasing dental floss. Numerous studies have demonstrated that household income inequality leads to decreased utilization of dental care services [27-29]. While dental floss is not prohibitively expensive, for households on tight budgets, even minor expenses can necessitate the prioritization of other essential needs over dental hygiene products. Second, economic status often correlates with educational level and health literacy [30]. Individuals with higher income levels generally have better access to education and are more likely to be aware of the benefits of flossing, the risks associated with poor oral hygiene, and the proper techniques for flossing [31]. Third, the issue of time poverty must also be considered. Those who work multiple jobs or long hours to make ends meet may find it challenging to dedicate time to oral healthcare, including daily hygiene practices [32]. For these individuals, the relatively quick act of flossing may be neglected in favor of other pressing daily concerns. Fourth, in lower-income communities, dental flossing may be perceived more as a luxury than a necessity [33,34]. This perception is exacerbated by a lack of targeted public health messaging that clarifies the importance of dental care and promotes flossing as an essential and accessible component of daily hygiene.

**Figure 2.**
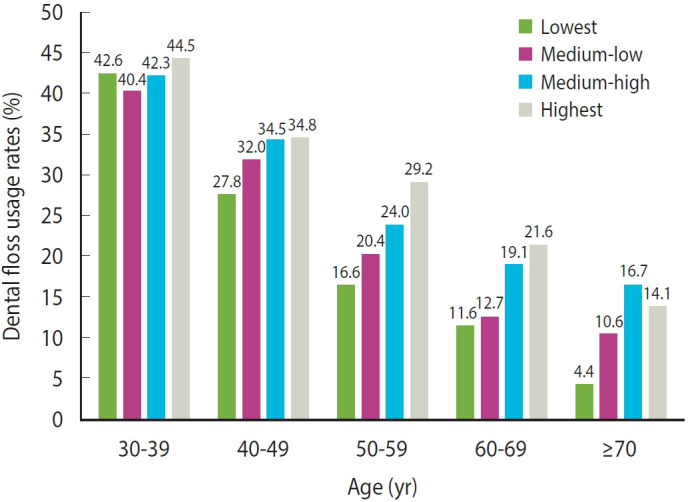
Household income-based differences in interdental brush usage among age groups.

As shown in Figure 2, the usage rate of dental floss was consistently higher among those with higher incomes across all age groups compared to those with the lowest incomes. Interestingly, for individuals aged 30-39 years and 40-49 years, the rate of flossing by income level was not significant. As age increased, a distinct pattern emerged where the disparity in flossing between income levels became more pronounced. In the analysis stratified by age groups, we observed an association between household income and flossing in 3 specific age groups: the 50s, 60s, and over 70s. The increasing prevalence of periodontitis with age has led to greater interest in oral hygiene products among older adults [35]. For elderly adults, limited productivity and unemployment can lead to poverty and income instability [36]. This may be a reason for the socioeconomic gradient observed.

In the gender-stratified analysis, the relationship between household income and flossing was evident in women but not in men. Epidemiological studies have shown that, irrespective of age, oral health behaviors in men are generally poorer than those in women [37,38]. A previous study demonstrated that women are more likely than men to seek regular dental care, and that higher income correlates with more frequent dental visits [39]. Therefore, it is reasonable to speculate that women, typically being more conscious of oral healthcare, are more inclined to pay for oral hygiene products like dental floss if they have higher incomes.

In the analysis stratified by periodontal health, individuals with periodontitis in the highest income brackets used dental floss more frequently than those in the lowest income brackets. Among the healthy population without periodontitis, those in the middle and highest income levels also used dental floss more often than those in the lowest income level. Managing gingivitis is the primary prevention of periodontitis [40]. Notably, the use of dental floss can alleviate the symptoms of gingivitis [41,42]. From this, we can infer that individuals without periodontitis often use dental floss to prevent periodontal health issues. Those with periodontitis may be following their periodontist or dentist’s advice to use dental floss during treatment [43,44]. The use of oral health supplements is a relatively inexpensive and easily accessible method, making it an important factor in promoting oral health and preventing oral health inequality [45,46]. However, our results show that flossing varies according to income. This variation may be due to individuals from lower socioeconomic backgrounds often having a poor self-perception of health and lacking health awareness [47]. Lastly, it can be speculated that individuals with periodontitis who have higher incomes are more aware of oral health and have greater financial means for care, leading to more frequent use of dental floss and other care products to limit the progression of periodontitis.

Our study has some limitations. First, as a cross-sectional study, it does not establish a causal relationship between household income and dental floss use. Additionally, the accuracy of the responses to our questionnaire may have been compromised by recall bias. Another significant limitation concerns our study population. The prevalence of severe periodontitis in elderly individuals might have been underrepresented due to the high incidence of edentulism and a significant number of missing teeth. Previous studies have highlighted the importance of having more than 20 teeth when used as an explanatory or outcome variable [48,49]. According to the KNHANES data, there was a notable difference in the prevalence of periodontitis based on the presence of more than 20 teeth among users of interdental cleaning devices. Consequently, groups with fewer than 20 teeth were excluded. Given this, it may be challenging to fully represent the actual situation, as those with poorer oral health, who are more likely to belong to lower-income groups, were not included. The primary aim of this study was to investigate the relationship between household income levels and flossing; therefore, educational level was not considered. Moreover, our study focused on Korean adults, limiting the generalizability of the results to other demographic groups, where different correlation patterns might be observed. Despite these limitations, the strength of this study lies in its use of a large national dataset, which enabled the analysis of a substantial number of samples. Our findings suggest the need for further public health initiatives in Korea. Future research should explore other aspects of oral health inequality, including studies with more inclusive criteria that encompass a broader spectrum of oral health conditions.

The results of this study highlight economic disparities in flossing among Korean adults. Individuals with higher incomes were found to floss their teeth significantly more often than those with lower incomes. This suggests that economic constraints prevent poorer individuals from engaging in this fundamental aspect of dental hygiene. These findings underscore the importance of considering economic factors in public health planning and the necessity for a more equitable allocation of health resources. Initiatives such as distributing dental care products in underserved communities or launching educational campaigns to enhance oral health awareness and promote better hygiene practices should be included in budget considerations.

## Figures and Tables

**Figure f3-epih-46-e2024052:**
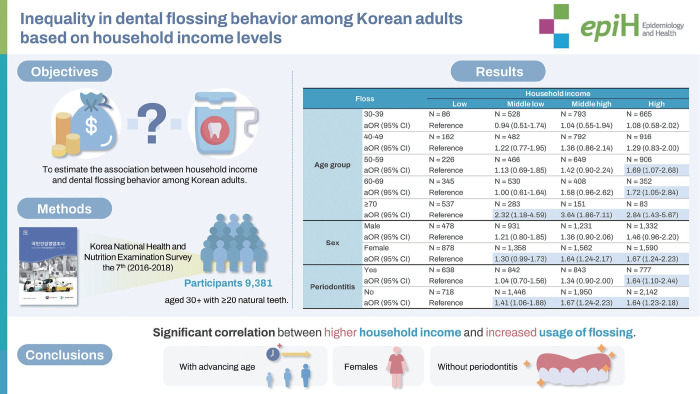


**Table 1.** Socio-demographic characterization of the study population according to their use of dental floss

Characteristics	Dental floss				p-value
	Yes		No		
Age, mean	2,528	46.4 (45.8, 47.0)	6,853	53.8 (53.3, 54.4)	<0.001
Gender					<0.001
Men	832	30.8 (29.1, 32.6)	3,151	43.9 (42.8, 43.9)	
Women	1,696	69.2 (67.4, 70.9)	3,702	56.1 (54.9, 57.2)	
Household income					<0.001
Lowest	175	7.1 (5.9, 8.6)	1,181	17.0 (15.5, 18.5)	
Medium-low	548	21.3 (19.2, 23.6)	1,741	25.1 (23.6, 26.7)	
Medium-high	841	33.1 (30.9, 35.4)	1,952	28.3 (26.9, 29.7)	
Highest	960	38.4 (35.5, 41.4)	1,962	29.6 (27.5, 31.8)	
Oral examinations during the past 1 yr					<0.001
No	1,275	50.3 (47.8, 52.7)	4,497	65.4 (63.9, 66.9)	
Yes	1,253	49.7 (47.3, 52.2)	2,356	34.6 (33.1, 36.1)	
Daily toothbrushing					<0.001
Once or none	397	15.1 (13.6, 16.8)	1,552	22.1 (20.9, 23.4)	
Twice or more	2,123	84.9 (83.2, 86.4)	5,230	77.9 (76.6, 79.1)	
Periodontitis					<0.001
No	2,013	80.2 (78.0, 82.2)	4,253	62.9 (61.0, 64.6)	
Yes	514	19.8 (17.8, 22.0)	2,597	37.1 (35.4, 39.0)	
Smoking					<0.001
Current	325	12.7 (11.1, 14.4)	1,306	18.0 (16.8, 19.2)	
Past	481	18.0 (16.4, 19.8)	1,529	22.0 (20.9, 23.1)	
Never	1,722	69.3 (67.3, 71.2)	4,017	60.0 (58.7, 61.3)	
Alcohol drinking					<0.001
No	193	7.4 (6.4, 8.5)	749	10.7 (9.9, 11.6)	
Yes	2,335	92.6 (91.6, 93.6)	6,102	89.3 (88.4, 90.1)	

Values are presented as unweighted number or weighted % (95% confidence interval).

**Table 2.** Analysis of the association between household income and flossing^1^

Household income	Model 1	Model 2	Model 3
n	9,360	9,302	9,302
Highest	1.92 (1.51, 2.44)^*^	1.65 (1.30, 2.11)^*^	1.63 (1.27, 2.08)^*^
Medium-high	1.69 (1.35, 2.13)^*^	1.59 (1.26, 2.00)^*^	1.57 (1.24, 1.99)^*^
Medium-low	1.37 (1.09, 1.73)^*^	1.32 (1.04, 1.67)^*^	1.31 (1.03, 1.65)^*^
Lowest	1.00 (reference)	1.00 (reference)	1.00 (reference)

Values are presented as odds ratio (95% confidence interval).

1 Model 1: adjusted for age and gender; Model 2: additionally adjusted for oral examinations during the past 1 year, daily toothbrushing, and periodontitis; Model 3: additionally adjusted for smoking and alcohol drinking.

* p<0.05.

**Table 3.** Association between household income level and flossing among subjects by age groups, gender, and periodontal status^1^

Floss	Household income			
	Lowest	Medium-low	Medium-high	Highest
Age				
30-39	1.00 (reference)	0.94 (0.51, 1.74)	1.04 (0.55, 1.94)	1.08 (0.58, 2.02)
40-49	1.00 (reference)	1.22 (0.77, 1.95)	1.36 (0.86, 2.14)	1.29 (0.83, 2.00)
50-59	1.00 (reference)	1.13 (0.69, 1.85)	1.42 (0.90, 2.24)	1.69 (1.07, 2.68)^*^
60-69	1.00 (reference)	1.00 (0.61, 1.64)	1.58 (0.99, 2.62)	1.72 (1.05, 2.84)^*^
≥70	1.00 (reference)	2.32 (1.18, 4.59)^*^	3.64 (1.86, 7.11)^*^	2.84 (1.43, 5.67)^*^
Gender				
Men	1.00 (reference)	1.21 (0.80, 1.85)	1.36 (0.90, 2.06)	1.46 (0.96, 2.21)
Women	1.00 (reference)	1.31 (0.99, 1.73)	1.64 (1.24, 2.17)^*^	1.67 (1.24, 2.23)^*^
Periodontitis				
Yes	1.00 (reference)	1.04 (0.70, 1.56)	1.34 (0.90, 2.00)	1.64 (1.10, 2.44)^*^
No	1.00 (reference)	1.41 (1.06, 1.87)^*^	1.66 (1.24, 2.23)^*^	1.64 (1.23, 2.18)^*^

Values are presented as adjusted odds ratio (95% confidence interval).

1 Confounder variables: age (≥30 yr), gender, oral examinations during the past 1 year, daily toothbrushing, periodontitis, smoking, alcohol drinking.

* p<0.05.
